# A spatial database of lowland cropping systems in Benin, Mali and Sierra Leone

**DOI:** 10.1016/j.dib.2019.103876

**Published:** 2019-03-26

**Authors:** Joel Huat, Elliott Dossou-Yovo, Moumini Guindo, Hermane Avohou, Théo Furlan, Fatogoma Sanogo, Amadou Touré

**Affiliations:** a CIRAD, UPR Hortsys, F-97600, Montpellier, France; b Africa Rice Center (AfricaRice), 01 BP 2551 Bouaké 01, Côte d’Ivoire; c Institut d'Economie Rurale (IER), BP 16, Sikasso, Mali; d University of Liège, Department of Pharmaceutical Sciences, Place du 20-Août, 7, 4000, Liège, Belgium; e ERABLES 31, 601 Route des Pyrénées, 31370 Poucharramet, France

## Abstract

This paper presents data collected in 2013, 2014 and 2015 on the cultural practices and agronomic performance of cropping systems in 500 lowland rice fields located in five regions of three West African countries, Benin, Mali and Sierra Leone. Data were collected in two stages. In the first stage, the main regions containing inland valleys were identified in each of the three countries and the most cultivated inland valley in each region was selected. Weather data were obtained from weather stations located close to the selected inland valleys. In regions with no weather stations, Tinytag data loggers were installed in the inland valleys to collect data on temperature, rainfall and relative humidity. In the second stage, the location and size of all the farmers' fields in each inland valley were determined using GPS devices. In 2013, soil samples were collected in each farmer's field and the soil physical-chemical properties were determined. Agronomic and socio-economic surveys were conducted to collect data on cultivated crops, crop sequences and management techniques using questionnaires and informal interviews. Crop yields were determined in each farmer's field in the growing season. The database contains a total of 131 variables divided into 9 themes: field characteristics, land preparation, field maintenance, irrigation, residue management, soil data, weather data, crop productions in the dry season and crop production in the rainy season.

**Table:** Specifications Table

Subject area	Agricultural Sciences, Social Sciences
More specific subject area	Food security, Agriculture
Type of data	Table (Excel format)
How the data were acquired	Face-to-face farmer surveys using questionnaires and informal interviews, geographic locations obtained with GPS devices, direct observations.
Data format	Raw, cleaned
Experimental factors	Not applicable
Experimental features	Not applicable
Data source location	The data were collected in 5 regions in 3 countries, see also Fig. 1. Benin, 2 regions 1. Mono 2. Couffo Mali, 1 region: 3. Sikasso Sierra Leone, 2 regions: 4. Bo 5. Kenema The geographic coordinates of each farmer's field are included in the data base.
Data accessibility	Data are provided with this article
Related research article	T. Furlan, R. Ballot, L. Guichard, J. Huat. Possible *ex-ante* assessment of rice-vegetable systems performances when facing data scarcity: use of the PERSYST model in West Africa. European Society for Agronomy. September 7th – September 10th, 2015, International Symposium for Farming Systems Design, 2015, Montpellier, France.

**Table:** 

**Value of the data** • Large multidisciplinary data set comprising 598 fields in 5 regions distributed in 3 countries in West Africa, including field characteristics, descriptions of land preparation, field maintenance, irrigation, residue management, soil, weather and crop productions in the dry and rainy seasons. • The data set can be used to map and characterize lowland cropping systems in West Africa [1], to analyze the long-term sustainability of lowland cropping systems, to assess the impact of climate change on lowland cropping systems, etc. • The data can be linked to spatial databases on soil nutrient levels [2], groundwater [3] and water quality to understand the ecological impacts of lowland cropping systems in Africa. • The current database is expected to form a background for the assessment of climate change impact on cropping systems in lowlands perceived as the future food baskets of Africa [4], [5].

## 1 Data

**Fig. 1:**
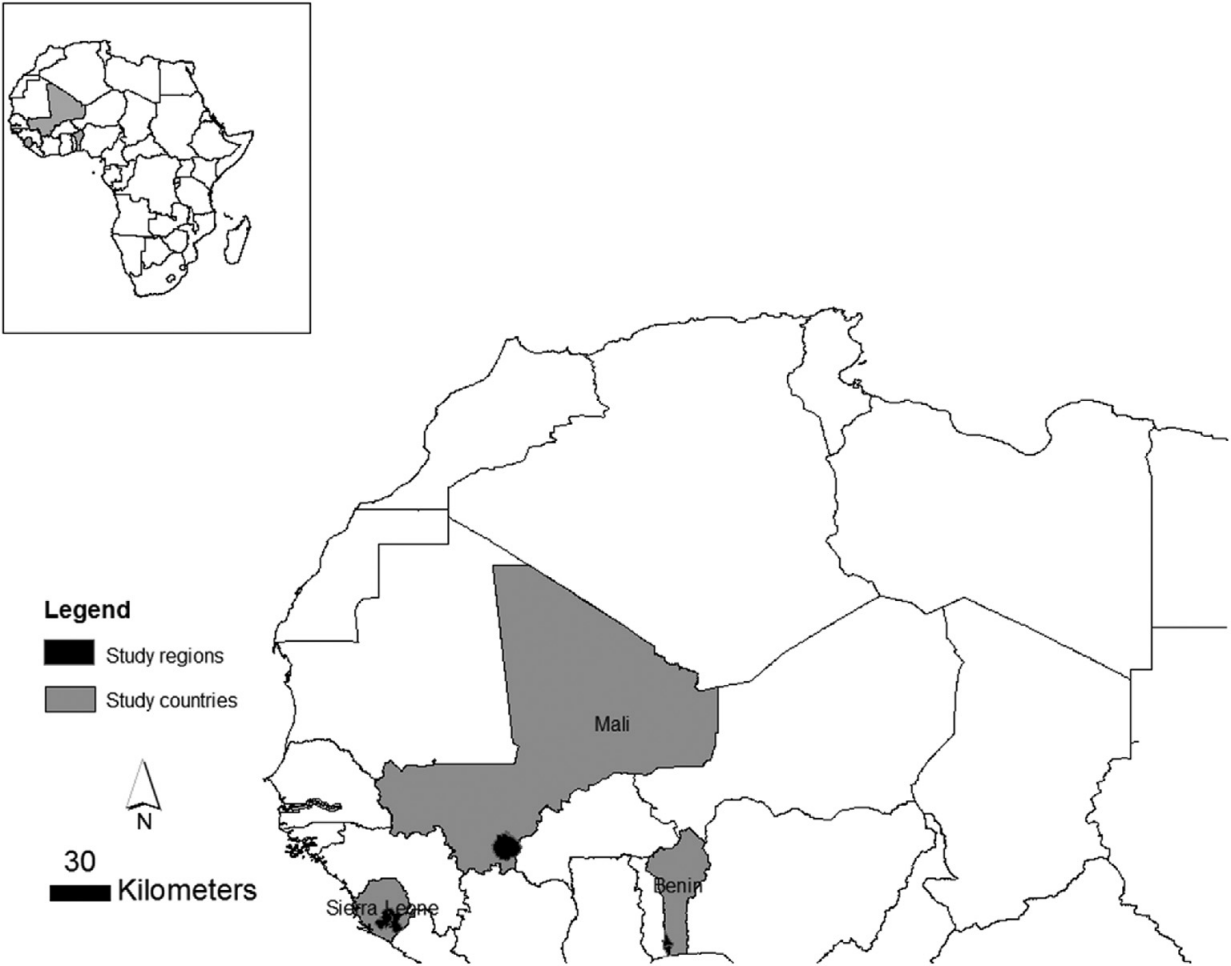
Location of the study areas in West Africa.

The database contains the location, weather, soil, crop sequence, management techniques, and yield data on 500 lowland rice fields located in five regions of three West African countries: Benin (227 lowland rice fields), Mali (173) and Sierra Leone (100) (see Fig. 1). The five regions cover three climate zones ranging from tropical humid (Bo and Kenema) in Sierra Leone to tropical sub-humid humid (Mono and Couffo) in Benin and sub-humid dry (Sikasso) in Mali. Each farmer's field is geolocated with latitude/longitude coordinates. For each farmer's field, 131 variables are grouped in 9 themes: field characteristics, land preparation, field maintenance, irrigation, residue management, soil data, weather data, crop production in the dry season and crop production in the rainy season (Table 1). Data were obtained from either farmers' responses during surveys conducted in the 2013, 2014 and 2015 growing seasons or from direct field observations and measurements. Table 1 summarizes the database and the variables it contains.

**Table 1:** Summary of the variables included in the database grouped by theme.

Variables	Scale type	Scale class	Source of data
**Theme 1: Field characteristics**			
Code to identify the field	Nominal	Unique code starting with the letter B for Benin, M for Mali and S for Sierra Leone. The letter is followed by an integer	surveys
GPS coordinates in decimal degrees	Numeric		surveys
Ecology of the field	Nominal	Lowland, upland	surveys
Location in the topo sequence	Nominal	Upper part, fringe, lower part of the topo sequence	surveys
Surface area of the field in ha	Numeric		surveys
**Theme 2: Land preparation operations**			
Code to identify the field	Nominal	Refer to field code in Theme 1	surveys
Code to identify the crop	Nominal	Name of crop in English	surveys
Cropping year	Numeric		surveys
Cropping season	Nominal	Cold dry season, warm dry season and rainy season	surveys
Type of land preparation	Nominal	Tillage, no-tillage, raised board, flat board	surveys
Period of land cleaning	Numeric	Number of the week in the year when the land was cleaned	surveys
Manpower used for cleaning	Numeric		surveys
Period of tilling the land	Numeric	Number of the week in the year when the land was tilled	surveys
Manpower used for tillage	Numeric		surveys
Period of land puddling	Numeric	Number of the week in the year when the land was puddled	surveys
Manpower used for puddling	Numeric		surveys
Period of land leveling	Numeric	Shouldn't the ‘Number of the week in the year when the land was levelled’ be included here?	surveys
Manpower used for land leveling	Numeric		surveys
Other complementary land preparation operations	Nominal	Nursery	surveys
Period of implementation of other operations	Numeric	Number of the week in the year when nursery was planted	surveys
Manpower used for other operations	Numeric		
**Theme 3: Field maintenance operations**			
Code to identify the field	Nominal	Refer to field code in Theme 1	surveys
Code to identify the crop	Nominal	Refer to crop code in Theme 2	surveys
Cropping year	Numeric		surveys
Cropping season	Nominal	Cold dry season, warm dry season and rainy season	surveys
Quantity of seed sown	Numeric		surveys
Method of sowing	Nominal	Pocket, broadcasting, transplanting, cuttings, direct sowing	surveys
Date of sowing	Nominal		surveys
Source of manure	Nominal	Rice straw; rice husks; poultry droppings; pig manure; other? manure; litter and compost	surveys
Quantity of manure used for first application	Numeric		surveys
Quantity of manure used for second application	Numeric		surveys
Date of first application of organic manure	Nominal		surveys
Date of second application of organic manure	Nominal		surveys
Manpower used for first application of organic manure	Numeric		surveys
Manpower used for second application of organic manure	Numeric		surveys
Number of organic manure applications	Numeric		surveys
Quantity of NPK supplied during first application	Numeric		surveys
Quantity of NPK supplied during second application	Numeric		surveys
Quantity of NPK supplied during third application	Numeric		surveys
Date of first application of NPK	Nominal		surveys
Date of second application of NPK	Nominal		surveys
Date of third application of NPK	Nominal		surveys
Formulation NPK fertilizer	Nominal		surveys
Manpower used for application of NPK fertilizer	Numeric		surveys
Quantity of urea supplied during first application	Numeric		surveys
Quantity of urea supplied during second application	Numeric		surveys
Date of first urea application	Nominal		surveys
Date of second urea application	Nominal		surveys
Manpower used for urea application	Numeric		surveys
Number of urea and NKP fertilizer applications	Numeric		surveys
Mode of fertilizer application	Nominal		
Other complementary operations aside from manure, pesticide and herbicide applications	Nominal	weeding	surveys
Date of first complementary operation	Nominal		surveys
Date of second complementary operation	Nominal		surveys
Manpower used for first complementary operation	Numeric		surveys
Manpower used for second complementary operation	Numeric		surveys
Quantity of herbicide applied in the field (mL)	Numeric		surveys
Date of herbicide application	Nominal		surveys
Commercial name of herbicide	Nominal		surveys
Active substance in herbicide	Nominal		surveys
Number of herbicide applications	Numeric		surveys
Manpower used for herbicide application	Numeric		surveys
Quantity of pesticide used to treat a field	Numeric		surveys
Date of first pesticide application	Nominal		surveys
Date of second pesticide application	Nominal		surveys
Date of third pesticide application	Nominal		surveys
Date of fourth pesticide application	Nominal		surveys
Commercial name of pesticide	Nominal		surveys
Active substance in pesticide	Nominal		surveys
Number of pesticide applications	Numeric		surveys
**Theme 4: Field irrigation operations**			
Code for field identification	Nominal		surveys
Field area	Numeric		surveys
Code to identify crops	Nominal		surveys
Cropping year	Numeric		surveys
Cropping season	Numeric		surveys
Period of irrigation	Numeric		surveys
Use of well as water source	Nominal	Yes, No	surveys
Use of drilling as water source	Nominal	Yes, No	surveys
Use of river as water source	Nominal	Yes, No	surveys
Use of another source	Nominal	Yes, No	surveys
Type of reservoir used for irrigation	Nominal	Calabash, pump and seal	surveys
Number of days of irrigation per month	Numeric	three times a week, twice a week, twice a day five days a week, twice a day four days a week, twice a day seven days a week	surveys
Volume of reservoir	Numeric		surveys
Mode of irrigation used	Nominal	Pocket and sprinkler	surveys
Duration of irrigation (h)	Numeric		surveys
Manpower used per irrigation event	Numeric		surveys
Total irrigated water	Numeric		surveys
Water quantity per irrigation event	Numeric		surveys
**Theme 5: Field residue management practices**			
Code to identify the field	Nominal		surveys
Code to identify the crop	Nominal		surveys
Cropping year	Numeric		surveys
Cropping season	Numeric		surveys
Date of harvest	Nominal		surveys
Crop residues from the field	Nominal	Yes, No	surveys
Crop residues used to feed animals	Nominal	Yes, No	surveys
Crop residues burned	Nominal	Yes, No	surveys
Crop residues incorporated in the soil	Nominal	Yes, No	surveys
Crop residues used for compost	Nominal	Yes, No	surveys
Crop residues abandoned	Nominal	Yes, No	surveys
Crop residues used for other purposes	Nominal	Yes, No	surveys
**Theme 6: Weather data**			
Daily rainfall (mm)	Numeric		Weather stations
Minimum daily temperature (°C)	Numeric		Weather stations
Maximum daily temperature (°C)	Numeric		Weather stations
Minimum daily relative humidity (%)	Numeric		Weather stations
Maximum daily relative humidity (%)	Numeric		Weather stations
**Theme 7: Soil data**			
Code to identify village	Nominal		Soil sampling and laboratory analysis
Code to identify field	Nominal		Soil sampling and laboratory analysis
Sampling period during the year	Nominal		Soil sampling and laboratory analysis
pH of water	Numeric		Soil sampling and laboratory analysis
Soil organic carbon (%)	Numeric		Soil sampling and laboratory analysis
Total nitrogen (%)	Numeric		Soil sampling and laboratory analysis
Available phosphorus (ppm)	Numeric		Soil sampling and laboratory analysis
Cation exchange capacity (meq/100g)	Numeric		Soil sampling and laboratory analysis
Exchangeable calcium (cmolc kg^−1^)	Numeric		Soil sampling and laboratory analysis
Exchangeable magnesium (cmolc kg^−1^)	Numeric		Soil sampling and laboratory analysis
Exchangeable potassium (cmolc kg^−1^)	Numeric		Soil sampling and laboratory analysis
Exchangeable sodium (cmolc kg^−1^)	Numeric		Soil sampling and laboratory analysis
Percentage of sand (%)	Numeric		Soil sampling and laboratory analysis
Percentage of silt (%)	Numeric		Soil sampling and laboratory analysis
Percentage of clay (%)	Numeric		Soil sampling and laboratory analysis
**Theme 8: Crop production in the dry season**			
Code to identify the field	Nominal		
Code to identify the crop	Nominal		
Cropping year	Numeric		
Cropping season	Numeric		
Number of plots	Numeric		4 m^2^ quadrat in the field
Plot surface area (m^2^)	Numeric		4 m^2^ quadrat in the field
Number of plants in a plot	Numeric		4 m^2^ quadrat in the field
Number of plants harvested per plot	Numeric		4 m^2^ quadrat in the field
Number of tubers	Numeric		4 m^2^ quadrat in the field
Number of non-perished tubers	Numeric		4 m^2^ quadrat in the field
Number of perished tubers	Numeric		4 m^2^ quadrat in the field
Total weight of harvested tubers (kg)	Numeric		4 m^2^ quadrat in the field
Weight of non-undamaged harvested tubers (kg)	Numeric		4 m^2^ quadrat in the field
Weight of undamaged harvested tubers (kg)	Numeric		4 m^2^ quadrat in the field
Number of broken tubers	Numeric		4 m^2^ quadrat in the field
Number of small caliber tubers	Numeric		4 m^2^ quadrat in the field
Weight of broken tubers (kg)	Numeric		4 m^2^ quadrat in the field
Weight of small caliber tubers (kg)	Numeric		4 m^2^ quadrat in the field
Weight of other crops except rice and potatoes (kg)	Numeric		4 m^2^ quadrat in the field
**Theme 9: Crop production in the rainy season**			
Code to identify the field	Nominal		
Code to identify the crop	Nominal		
Cropping year	Numeric		
Cropping season	Numeric		
Number of plots	Numeric		4 m^2^ quadrat in the field
Number of plants at 20 days after sowing	Numeric		4 m^2^ quadrat in the field
Number of plants at 75 days after sowing	Numeric		4 m^2^ quadrat in the field
Number of panicles per plot	Numeric		4 m^2^ quadrat in the field
Average height of plants at maturity per plot (m)	Numeric		4 m^2^ quadrat in the field
Number of grains per panicle	Numeric		4 m^2^ quadrat in the field
Average percentage of whole grain (%)	Numeric		4 m^2^ quadrat in the field
Average 1000 grain weight (kg)	Numeric		4 m^2^ quadrat in the field

The database is in Microsoft Excel format and contains eleven sheets. The first sheet (Variables description) provides an explanation of the variables. The second sheet (VILLAGE) contains the names of lowlands investigated, the names of the villages, and regions in which the lowlands are located. The third sheet (FIELD) contains the list of fields cultivated by each farmer, their geolocation and surface area. The fourth sheet (FIELD PREPARATION) describes all land preparation operations, the period the operations were undertaken and the manpower allocated to each farmer's field. The fifth sheet (FIELD MAINTENANCE) describes planting, crop maintenance operations (manuring, weeding and pesticide application) and manpower allocated for all the operations implemented in each farmer’ field. The sixth sheet (FIELD IRRIGATION) describes irrigation operations including methods, frequency and the amount of water supplied. The seventh sheet (FIELD RESIDUES) contains the quantity of residues exported, left in the field or used to feed livestock for each farmer's field. The eighth sheet (WEATHER) contains daily weather data (temperature, relative humidity and rainfall) from 2013 to 2015 concerning the inland valley in which the village is located. The ninth sheet (FIELD SOIL ANALYSES) contains data on soil physical-chemical characteristics (particle size distribution, pH of the water, organic carbon, total nitrogen, available phosphorus, total potassium, cation exchange capacity, exchangeable calcium, magnesium and sodium) for each farmer's field. The tenth sheet (PLOT FIELD CS) contains yield data measured in each farmer's field in the 2013, 2014 and 2015 dry seasons. The eleventh sheet (PLOT PROD HIV) contains yield data measured in each farmer's field in the 2013, 2014 and 2015 rainy seasons.

Many values are missing in the tables for different reasons: data were not collected or we were not able to collect them, data were not viable after checking, no agronomic measurements were done or no technical operation was done in the field by the farmers.

## 2 Experimental design, materials and methods

This section provides a summary of the methods used to create the database. Data were collected in two stages. In the first stage, the main regions containing inland valleys in three West African countries viz. Benin, Mali and Sierra Leone were identified and the most cultivated inland valley in each region was selected. Weather data were collected from weather stations located close to the inland valleys concerned. In regions with no weather stations, Tinytag data loggers were installed in each of the selected inland valleys and used to record daily data on temperature, rainfall and relative humidity. In the second stage, the location and surface area of all the farmers' fields in each inland valley were determined with handheld GPS devices. In 2016, soil samples were collected in each farmer's field and the soil physical-chemical properties were determined. Socio-economic surveys were conducted from 2013 to 2015 to collect data on farmers' crops, crop sequences and management techniques using questionnaires and informal interviews. Crop yields were determined in 4 m^2^ quadrats in each farmer's field in the 2013, 2014 and 2015 growing seasons. Table 1 gives an overview of the 131 variables in the database and their source (surveys, weather stations, soil sampling and laboratory analyses or direct field observations and measurements).
